# Augmented Reality in Real-time Telemedicine and Telementoring: Scoping Review

**DOI:** 10.2196/45464

**Published:** 2023-04-18

**Authors:** Alana Dinh, Andrew Lukas Yin, Deborah Estrin, Peter Greenwald, Alexander Fortenko

**Affiliations:** 1 Medical College Weill Cornell Medicine New York, NY United States; 2 Department of Internal Medicine Weill Cornell Medicine New York, NY United States; 3 Department of Computer Science Cornell Tech New York, NY United States; 4 Emergency Medicine NewYork-Presyterian Hospital New York, NY United States

**Keywords:** augmented reality, telemedicine, telehealth, telementoring, teleguidance, telecommunication, teleconsultation, telecollaboration, scoping review, mobile phone

## Abstract

**Background:**

Over the last decade, augmented reality (AR) has emerged in health care as a tool for visualizing data and enhancing simulation learning. AR, which has largely been explored for communication and collaboration in nonhealth contexts, could play a role in shaping future remote medical services and training. This review summarized existing studies implementing AR in real-time telemedicine and telementoring to create a foundation for health care providers and technology developers to understand future opportunities in remote care and education.

**Objective:**

This review described devices and platforms that use AR for real-time telemedicine and telementoring, the tasks for which AR was implemented, and the ways in which these implementations were evaluated to identify gaps in research that provide opportunities for further study.

**Methods:**

We searched PubMed, Scopus, Embase, and MEDLINE to identify English-language studies published between January 1, 2012, and October 18, 2022, implementing AR technology in a real-time interaction related to telemedicine or telementoring. The search terms were “augmented reality” OR “AR” AND “remote” OR “telemedicine” OR “telehealth” OR “telementoring.” Systematic reviews, meta-analyses, and discussion-based articles were excluded from analysis.

**Results:**

A total of 39 articles met the inclusion criteria and were categorized into themes of patient evaluation, medical intervention, and education. In total, 20 devices and platforms using AR were identified, with common features being the ability for remote users to annotate, display graphics, and display their hands or tools in the local user’s view. Common themes across the studies included consultation and procedural education, with surgery, emergency, and hospital medicine being the most represented specialties. Outcomes were most often measured using feedback surveys and interviews. The most common objective measures were time to task completion and performance. Long-term outcome and resource cost measurements were rare. Across the studies, user feedback was consistently positive for perceived efficacy, feasibility, and acceptability. Comparative trials demonstrated that AR-assisted conditions had noninferior reliability and performance and did not consistently extend procedure times compared with in-person controls.

**Conclusions:**

Studies implementing AR in telemedicine and telementoring demonstrated the technology’s ability to enhance access to information and facilitate guidance in multiple health care settings. However, AR’s role as an alternative to current telecommunication platforms or even in-person interactions remains to be validated, with many disciplines and provider-to-nonprovider uses still lacking robust investigation. Additional studies comparing existing methods may offer more insight into this intersection, but the early stage of technical development and the lack of standardized tools and adoption have hindered the conduct of larger longitudinal and randomized controlled trials. Overall, AR has the potential to complement and advance the capabilities of remote medical care and learning, creating unique opportunities for innovator, provider, and patient involvement.

## Introduction

### Background

Augmented reality (AR) is an emerging technology that can enhance how the real world is experienced by the user. Compared with virtual reality (VR), in which the user is immersed in a completely synthesized world, AR combines both the virtual and real by overlaying the external world with computer-generated sensory data such as audio, video, and graphics. AR technology, often accessed through head-mounted devices (HMDs) or software on personal devices, can be used to display information and virtual objects that facilitate learning and navigation through tasks in the real world [1,2].

In medicine, VR and AR have been explored in educational, diagnostic, and treatment settings, with an increasing number of publications in the last decade [3-6]. A 2012 to 2017 review of 338 original studies using AR in medicine, most related to surgery and simulation learning, estimated the technology readiness level of AR to be at the stage of a prototype that has yet to be completed and tested in its intended environment [7]. AR has since appeared in the literature across many specialized fields, with reviews since 2019 describing AR technology in emergency medicine (EM) [8], dermatology [9], radiology [10,11], orthopedics [12], nursing [13], and many more.

A promising use of AR technology is in remote collaboration, an application seen in many industry- and engineering-related tasks over the last 2 decades [14,15]. As COVID-19 pushed health care to explore remote health solutions, providers, caregivers, and students have become increasingly aware of technology’s role in enabling access to care [16-18]. A cohort study of 36.5 million individuals in the United States found that 23.6% of ambulatory visits in 2020 were billed as telehealth visits compared with 0.3% in 2019 [19]. Although videoconferencing programs allow health care workers to remotely connect with each other, trainees, and patients, current systems limit the extent of care that can be delivered during such interactions [20]. Innovations using AR technology offer an opportunity to expand real-time remote health services such as consultation and telesurgery [21]. Recent literature on AR includes studies on caregiver perspectives on the technology [22], frameworks for AR-assisted remote medical communication [23], and remote health care delivery devices incorporating AR [24].

### Objectives

Remote medical communication is a defining feature of *telemedicine*, a concept that first arose with the use of telephones to share information across hospital systems [25]. Since the conception of the internet and personal devices, remote health care visits and interventions have become possible, with the scope of care expanding as technological advances are introduced. Currently, there is no literature summarizing the applications of AR in *synchronous telemedicine*, defined as the use of electronic devices for real-time communication in health care services related to patient encounters, treatment, and consultation [25,26]. *Telementoring*, a subcategory of telemedicine, is the real-time remote guidance of health care procedures or skills. Telementoring also plays a role in granting remote access to care and expertise, enabling the sharing of specialized knowledge and education [27]. Similar to telemedicine, it is also a topic scarcely studied in relation to AR and has the potential to evolve with technological innovation [28]. By exploring how AR is used for real-time telemedicine and telementoring, this review could better inform health care providers and developers of AR’s future potential and current limitations.

## Methods

### Scoping Method

Scoping reviews entail a systematic selection of literature with the purpose of examining the extent and nature of an area of interest [29,30]. Compared with systematic reviews, scoping studies allow for the integration of a range of study designs, especially in fields with emerging evidence that may lack randomized controlled trials (RCTs). By mapping the existing evidence of AR in telemedicine, the scoping approach allows for the identification of gaps that may inform future studies and innovations.

### Research Questions

Which devices and platforms using AR have been studied in the published literature in the context of real-time telemedicine and remote education? In which areas of medicine have these been integrated and for what purposes? How are outcomes evaluated and what variables have yet to be measured? What are the overall findings of existing studies?

### Identifying Studies in the Literature

The literature was reviewed using PubMed, Scopus, Embase, and MEDLINE for articles or trials published from January 1, 2012, to October 18, 2022, with search queries submitted and articles accessed on October 18, 2022. The search terms were “augmented reality” OR “AR” AND “remote” OR “telemedicine” OR “telehealth” OR “telementoring.” The PubMed search was performed using article titles and abstracts. The Scopus search was performed using article titles, abstracts, and keywords, with articles and conference papers in the areas of “medicine,” “health professions,” and “nursing” included. The Embase search was performed using article titles, abstracts, and keywords, with articles and conference papers included. The MEDLINE search was also performed using article titles, abstracts, and keywords. Only articles available in English were included.

### Article Selection

Following the PRISMA (Preferred Reporting Items for Systematic Reviews and Meta-Analyses) guidelines, the titles and abstracts of all articles were independently reviewed by 2 researchers (AD and AF) for relevance to AR and real-time communication between separated individuals. Articles were excluded if they were unrelated to medicine or were reviews or discussions. Any articles that were included by one reviewer but not the other were included in the full-text screening, which was also performed independently by the 2 researchers. As this review focused on the use of AR rather than the development of related equipment or software, articles were excluded if the technological design, rather than the implementation, was the focus or if the technology was not intended for remote interaction as described by the inclusion criteria. Articles that included both technological design and implementation data were included, with the review focusing on the latter. Articles that described mixed reality devices capable of both AR and VR were included if the implementation primarily used and studied AR features. Reviews, perspectives, discussion-based articles, and study proposals without results were excluded. Correspondence was sent to the authors of articles for which the full text was not available; a lack of response resulted in the exclusion of these articles. Any disagreements regarding an article’s inclusion were resolved through discussion between the 2 reviewers.

### Data Charting

The articles were reviewed based on the context in which the AR was implemented. Articles describing mixed reality devices were analyzed for data relevant to AR use and not VR. Unique devices and platforms using AR across the articles were identified. The articles were later grouped into one of the 3 identified areas: patient evaluation, medical intervention, and education. “Patient evaluation” included articles that described the examination of patients and processes that obtained information for clinical decision-making. “Medical intervention” included articles that described procedures related to the initiation or provision of therapy. “Education” included articles that described the mentorship or training of a less experienced individual for a task or procedure. Subgroups for surgical versus nonsurgical tasks in the latter 2 groups were created. Articles that fell into more than one of the 3 identified areas were organized in the Results section of this paper based on which area was the primary focus. Finally, common objective and subjective end variables discussed in the articles were identified.

### Collation and Summary

For this scoping review, we first provide an overview of the devices and platforms that use AR and the types of tasks in which they appear. We then summarize the implementation and measurement of these AR-capable tools within 3 areas: patient evaluation, medical intervention, and education. Finally, we review the common methodologies and end variables observed across the studies.

## Results

### Overview

**Figure 1:**
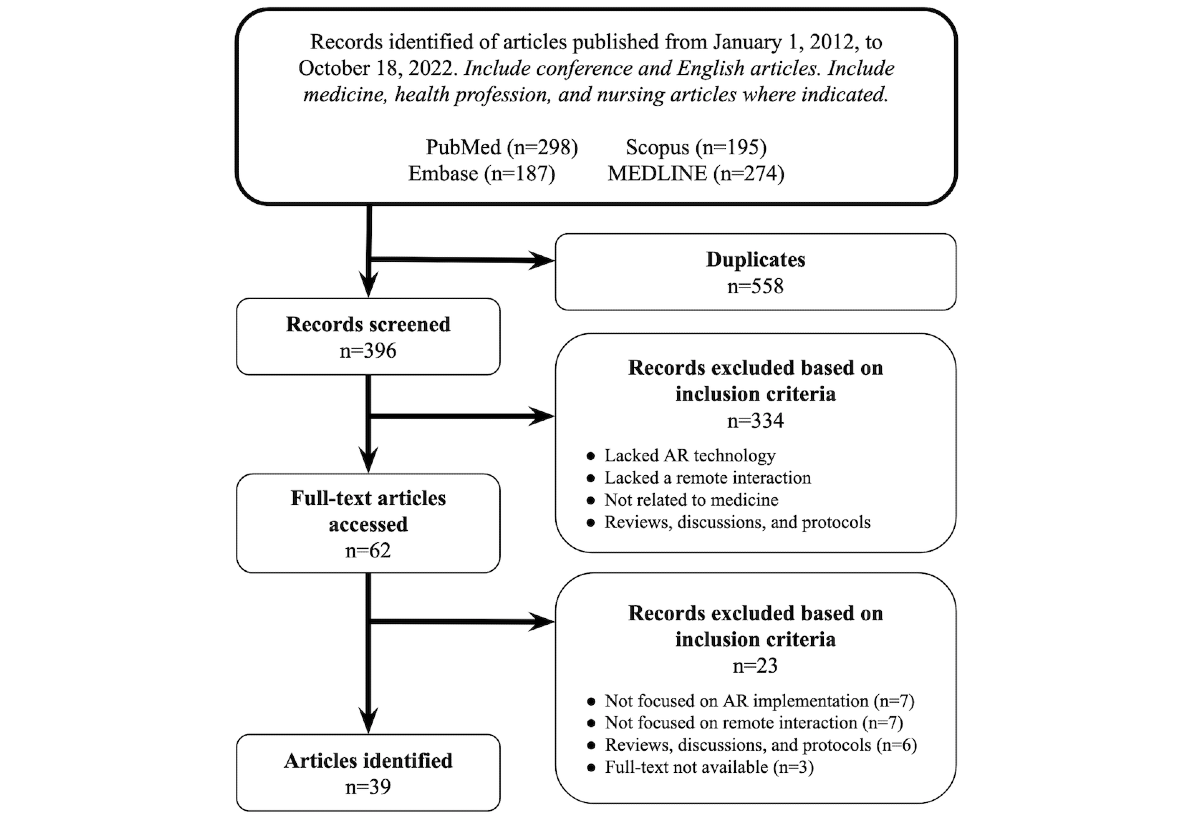
Selection process for the articles. AR: augmented reality.

The PubMed, Scopus, Embase, and MEDLINE searches yielded 298, 195, 187, and 274 articles, respectively. This totaled 954 articles, 558 (58.5%) of which were identified as duplicates. The abstracts and titles of the remaining 396 articles were reviewed, with 62 (15.7%) identified as meeting the inclusion criteria. In total, 5% (3/62) of the articles, for which the full text was not available, were excluded after no response was received from the original authors. A total of 39 articles were included following full-text screening. The selection process is depicted in Figure 1. The publication years of the selected articles spanned 2014 to 2022, with 64% (25/39) published in the last 3 years.

From the included articles, 20 unique devices and platforms with AR features were identified, with 10 (50%) being commercial HMDs in which AR features were projected before the wearer’s eyes and another 4 (20%) being “virtual presence”–type platforms in which a remote viewer can superimpose video of their hand or tools over the live stream of a local site or procedure; the hybrid video is accessed by both local and remote users via a smartphone, tablet, computer, or monitor. The remaining 30% (6/20) involved systems with no commercial HMDs or virtual presence—these included a smartphone app and systems built specifically for tele-ultrasonography, physical rehabilitation, and surgical telementoring. Overall, all the identified devices and platforms (20/20, 100%) allowed remote individuals to view the perspective or environment of the local user. Common AR features of these devices included annotation (12/20, 60%) and graphical overlay over the local user’s view, specifically 2D or 3D images (9/20, 45%) and the remote viewer’s hands or tools (8/20, 40%). The identified devices and platforms are listed in Table 1, with similar device models included in the same row.

**Table 1:** Overview of devices and features.

Tool (year)		Communication features	Visual features	Relevant studies
**Commercial HMDs^a^**				
	Vuzix Wrap 920AR (2010)	Internet transmission of video data	Camera-captured video feed of mentor’s hand gestures is overlaid on mentee’s HMD, and vice versa	Chinthammit et al [31]
	Vuzix Wrap 1200DX (2013)	2-way audio-video communication over Wi-Fi	Camera-captured images of mentor’s hand gestures are transmitted to mentee’s HMD	Mather et al [32]
	Recon Jet (2013)	2-way audio communication over phone or Wi-Fi Integration with custom software for 1-way video feed to computer	Integration with custom Android-based triage app	Follmann et al [33]
	Google Glass (2013-2017)	Compatible with Google Hangouts for audio-video streaming on remote devices and 2-way audio communication	Projects SMS text messages from remote viewers into local user’s view Remote viewer moves a mouse cursor in local user’s view Remote viewer’s webcam captures image of their hands or tools that superimpose onto local user’s view	Broach et al [34] Ponce et al [35] Armstrong et al [36]
	Microsoft HoloLens (2016)	Compatible with Skype or other Windows applications for audio-video streaming on remote devices and 2-way audio communication	Displays instructions, patient data, and images Remote viewers annotate local user’s view and create 3D surgical trajectories on virtual limbs Displays hand gestures captured by sensor device used by remote mentor	Kaylor et al [37] Cofano et al [38] Liu et al [39] Hanna et al [40] Wang et al [41]
	Moverio BT-300 (2016) and Moverio BT-350 (2017)	Compatible with TeamViewer app for audio-video streaming on remote devices and 2-way audio communication	Remote viewers directly annotate local user’s view Remote viewers create and annotate screenshots to be displayed in local user’s view	Cofano et al [38]
	Vuzix Blade (2018)	Compatible with TeamViewer app for audio-video streaming on remote devices and 2-way audio communication	Remote viewers directly annotate local user’s view Remote viewers create and annotate screenshots to be displayed in the local user’s view	Cofano et al [38]
	Magic Leap One (2018)	2-way audio communication over Wi-Fi	Displays holographic patients and monitors that can be modulated by remote viewers	Hess et al [42]
	Microsoft HoloLens 2 (2019)	Compatible with Microsoft Teams for audio-video streaming on remote devices and 2-way audio communication	SMS texts messages from remote viewers projected onto local user’s view Display patient data and images Remote viewers directly annotate and blend pictures or videos into local user’s view 3D object annotation	Hill [43] Rigamonti et al [44] Martin et al [45] Van der Putten et al [46] Rafi et al [47] Bala et al [48] Mill et al [49]
**Virtual presence tools**				
	Original augmented reality telementoring platform (2014)	Wired connection allows for the sharing of video feeds and audio communication	Image of mentor’s laparoscopic instruments is superimposed onto mentee’s monitor; hybrid video seen at both sites	Vera et al [50]
	Virtual interactive presence and augmented reality platform (2013)	2-way video streaming via internet Can combine with Skype or other teleconferencing software	Remote viewer’s hand or instrument is superimposed over local video feed Remote viewer can freeze screen or 2D annotate image using pen tool	Ponce et al [51] Ponce et al [35] Vyas et al [52] Davis et al [53]
	Help Lightning mobile app (2016)	2-way internet transmission of audio and video between phones	Foreground of physician’s video (eg, physician’s hand) is superimposed over patient’s video for live gesturing Physician annotates over patient’s live video	Ponce et al [54]
	Proximie (2016)	Audio-video streaming from local site can be accessed by remote computers via internet Viewers from different remote sites can access the same live stream and talk with each other and the local site	Virtual hand pointer or pen to mark video feed from local site Image of remote viewer’s hand is superimposed over video from local site Overlaying video with 2D images and 3D models Computer vision algorithm allows for the anchoring of annotations	Hassan et al [55] El-Asmar et al [56] Greenfield et al [57] Patel et al [58]
**Other original systems**				
	System for Telementoring with Augmented Reality platform (2015)	2-way internet transmission of audio and video between tablets or to HMD	Remote viewer annotates over local site’s video Remote viewer places scalable instrument icons or labels over local site’s video Local user can access prerecorded video clips to guide procedure in the event that the remote connection is unstable [59]	Rojas-Muñoz et al [60-62] Andersen et al [59,63]
	Vuforia Chalk mobile app (2017)	2-way internet transmission of audio between devices Internet transmission of camera feed from local user to remote expert	Remote viewer annotates over local user’s live video Annotations remain anchored to objects in the video even if local camera moves	Ramsingh et al [64]
	Original tele-ultrasound system (2018)	Open-source software for communication between remote viewer and local user Internet transmission of ultrasound video and live video of local user’s environment sent to remote viewer’s laptop	Remote viewer draws or writes directly on ultrasound images being streamed by local user; hybrid video seen at both sites	Carbone et al [65]
	Augmented Reality–based Telerehabilitation System with Haptics (2019)	2-way internet transmission of audio and visual data via computers Haptic devices relay force feedback and motion to each other through networked computer	Camera data used to generate image of remote and local users sitting across from each other in a virtual space seen on 3D televisions	Borresen et al [66,67]
	Telestration with coaxial projective imaging (2022)	2-way audio-video streaming over the internet	Obtains images from local field that remote viewer can annotate System projects annotations onto local field directly	Zhang et al [68]
	Original remote training platform (2022)	2-way audio-video streaming over the internet	HMD optics has see-through transparency and enables display of instructional information and video	Stone et al [69]

^a^HMD: head-mounted device.

Of the 39 studies, 22 (56%) were related to surgery, 6 (15%) were related to EM, and 4 (10%) were related to hospital medicine. When looking at the setting and structure of the tasks from each study, 51% (20/39) involved an operation or technique used in the operating room, where a mentor figure used AR to remotely interact with a task performer. The remaining 19 studies involved nonoperative tasks, 11 (58%) of which also involved a mentor remotely interacting with a task performer, whereas 8 (42%) involved the mentor performing the task instead, with AR enhancing what their remote spectators saw.

The articles were divided into 3 sections based on the AR-assisted task performed: patient evaluation, medical intervention, and education (Multimedia Appendix 1-3). Medical intervention and education are further subdivided based on whether AR supported a nonsurgical or surgical task. Notably, some articles discussed in the medical intervention section were also relevant to education.

### AR in Remote Patient Evaluation

The 26% (10/39) of articles included in this section (Multimedia Appendix 1 [33,34,37,43-45,54,65-67]) described implementations of AR in remote triage, wound assessment, musculoskeletal examination, sonography, and hospital rounding.

The potential of AR to perform fast-paced triage assessments remotely was explored in 20% (2/10) of the studies. Broach et al [34] investigated the use of Google Glass to relay what paramedics saw to other remote providers, with the intention of allowing the remote EM physicians to perform secondary triage before the patient’s arrival at a hospital. The remote physicians accessed the perspective of the paramedics at a simulated disaster scene and could send instructional SMS text messages projected onto the paramedics’ Glass. When comparing the remote assessments of physicians with the in-person assessments by different EM physicians, the study found high interrater agreement (0.923), which was not significantly different from the interrater agreement within the same assessment condition (0.976; *P*=.41) [34]. Follmann et al [33] investigated how AR implementation could affect triage time and accuracy. The performance of non–AR-assisted first responders was compared with that of 2 groups using AR-capable glasses, one that displayed an interactive triage algorithm and the other that streamed footage to a remote EM physician who could verbally guide the on-site individual. The results revealed that AR assistance increased accuracy at the cost of time, with the accuracy of the 3 groups being 58%, 92% (*P*=.04), and 90% (*P*=.01), whereas the duration of triage was 16.6, 37.0 (*P*=.001), and 35.0 (*P*=.01) seconds, respectively [33].

Other studies focused on AR’s potential to enhance remote wound assessments. Ponce et al [54] demonstrated the use of AR with mobile devices to allow orthopedic surgeons and neurosurgeons to conduct remote postoperative visits. Using a virtual interactive presence (VIP) smartphone app, surgeons overlaid live camera footage of their hands over the camera footage of the patient’s postoperative wound. Surveys from users revealed that both patients and surgeons found utility (27 of 28 and 26 of 29 with positive responses) in the virtual experience [54]. Kaylor et al [37] and Hill [43] focused on the use of AR for consultations during inpatient wound assessments. Using Microsoft HoloLens, bedside nurses in the former study could send live video of a patient’s wound and communicate with a remote wound, ostomy, and continence (WOC) nurse. The remote WOC nurse could provide annotations or images to guide the local nurse during the assessment. When comparing remote assessments with in-person assessments performed by a different WOC nurse, the study found the interrater agreement of treatment plans to be 100% across 21 cases [37]. The bedside nurses in the study by Hill [43] used the Microsoft HoloLens 2 to consult for complications at night or over weekends for patients undergoing negative pressure wound therapy. Compared with a control group of previous cases that did not use AR, the study group underwent fewer unplanned surgical revisions (*P*=.002) and admissions related to wound infection (*P*=.004) [43].

Borresen et al [66,67] introduced the AR-based Telerehabilitation System with Haptics (ARTESH) to perform remote strength and range-of-motion examinations. Both the local and remote sites were equipped with a haptic device, a Kinect camera, and a 3D-capable television that allowed the physician and patient to view each other seated together at a virtual table. The 7-point Likert scale surveys from the pilot study showed positive ratings from 5 physicians on the ability to evaluate arm strength and visualize limb movement (6/7 and 5.87/7) [66]. A follow-up study measured interrater agreement between in-person examinations and remote evaluations using ARTESH for different components of the upper extremity examination. The highest levels of agreement were observed in strength testing of elbow flexion, shoulder abduction, and protraction (κ=0.63, 95% CI 0-1.0), with the percentage of interrater agreement across evaluations for all 15 patients ranging from 30 to 100 [67].

Rigamonti et al [44] and Carbone et al [65] conducted studies that integrated AR technology to allow for supervision and consultation while performing ultrasound examinations. Rigamonti et al [44] interviewed engineering and sports science professionals across 6 countries after they accessed live video footage from a Microsoft HoloLens 2 worn by an ultrasound operator in Germany. Users were able to annotate the stream, overlay pictures and videos onto the display, and communicate in real time as they watched the examination from the operator’s perspective. Interview responses from the spectators revealed AR’s potential in both education and enhancing remote examinations with the supplementation of live data and feedback [44]. Carbone et al [65] developed a tele-ultrasound system that allowed rural hospital clinicians to contact a consultant. The remote consultant, who viewed live video of the user’s environment and ultrasound sequences, could provide audio feedback and overlay annotations or a cursor on the ultrasound imaging seen at the local site. Although the connected parties primarily used the platform to discuss the diagnosis, in 5 of 12 cases, the consultant directed the user on the device’s probe position [65].

The study by Martin et al [45] used an AR-capable Microsoft HoloLens 2 in multiple COVID-19 wards. The senior member of a clinical team would wear the headset and personal protective equipment (PPE) while examining patients, allowing the remaining members of the team to watch and interact remotely. Viewers could remotely annotate objects as well as overlay patient imaging and data from the electronic health record onto the view of the headset user and the live video feed. In comparison with teams without the device, the AR-assisted teams saw less COVID-19 exposure time by 51.5% and decreased PPE use by 83.1% over a week (*P*=.002 and *P*=.02) [45].

### AR in Remote Medical Intervention

The 41% (16/39) of articles included in this section (Multimedia Appendix 2 [31,35,38,39,46,52,53,55-57,59-61,63,64,68]) described AR in remote nonsurgical and surgical contexts. The latter included studies that focused on surgical efficiency, long-distance consultation, and differences between telesurgical systems.

#### Nonsurgical

Chinthammit et al [31] introduced the “Ghostman” system, in which a patient and a remote physical therapist are connected via AR-capable headsets (Vuzix Wrap 920AR). The hands and tools of the therapist were overlaid onto the patient’s headset, allowing the patient to obtain real-time feedback during the session. To test the system’s potential for telerehabilitation, an RCT was designed with 2 groups of volunteers receiving training on how to use chopsticks, one group with AR assistance and the other with face-to-face mentoring. Assessments performed immediately after, 1 day after, and 7 days after training found no significant difference in total skill errors or time to task completion between the 2 groups [31].

Ramsingh et al [64] described the use of a commercial smartphone app (Vuforia Chalk) to allow experts in Loma Linda, California, United States, to support an ultrasound-guided popliteal nerve block performed in Port-au-Prince, Haiti. The remote expert viewed the patient and ultrasound monitor through the local smartphone’s camera and created annotations that appeared on the local smartphone screen to guide the procedure. Both local and remote users rated the quality of the video communication as 5/5, whereas the local user rated the clarity of the AR annotations in guiding probe placement as 5/5 and the identification of relevant anatomy in ultrasound imaging as 4/5 [64].

#### Surgical

The efficacy of various AR-capable tools in the operative setting was tested in 25% (4/16) of the studies. Rojas-Muñoz et al [60] designed an RCT comparing audio-only telementoring against the System for Telementoring with AR (STAR) combined with an HMD (HMD-STAR) in the setting of emergency cricothyroidotomies by first responders. HMD-STAR use, in which a remote mentor placed helpful annotations and icons in the responder’s line of sight, increased performance scores when considering all-experience groups (*P*=.01) and those with low first-responder experience (*P*=.01) and low procedure experience (*P*=.03) [60]. Cofano et al [38] surveyed orthopedic surgeons who used various AR-capable HMDs for telementoring and visualization of 3D anatomical reconstructions. The surgeons reported positive feedback on the ergonomy of the headsets and perceived them as a beneficial tool that would shorten procedures and reduce postoperative complications [38]. Hassan et al [55] and El-Asmar et al [56] described neurointerventional and urological case series in which the telesurgery platform Proximie was used. Proximie allows a remote surgeon to overlay live video of their hands and tools onto a live stream of the operating field, thereby giving the consulting surgeon, who sees the hybrid video on a monitor in the operating room, real-time guidance. Hassan et al [55] observed no complications after 10 neurovascular procedures and noted no significant difference in contrast dye use or fluoroscopy times compared with similar on-site procedures (*P*=.38 and *P*=.85) [55]. El-Asmar et al [56] compared 21 AR-proctored aquablation procedures with 38 on-site guided cases and found no significant difference in length of stay, hospitalization, and 3-month adverse events [56].

Other studies (6/16, 38%) used AR in long-distance consultation and global surgery. Greenfield et al [57] described a case report in which a surgeon in Gaza, Palestine, connected with a specialist in Beirut, Lebanon, via Proximie for a hand reconstruction procedure. Ponce et al [35] described a case report that used VIP and AR (VIPAAR) to connect a remote consultant in Atlanta, Georgia, with a Google Glass–wearing orthopedic surgeon in Birmingham, Alabama, for a successful shoulder replacement. Similar to Proximie, VIPAAR allows the remote user, equipped with a computer and web camera, to create annotations and superimpose video of their hands or instruments over the local video of the operating field. However, instead of a hybrid video appearing on a monitor at the local site, the manipulations of the remote viewer would appear on the Glass worn by the local surgeon. Vyas et al [52] and Davis et al [53] focused on VIPAAR for telesurgery across continents. Vyas et al [52] described surgeons in Peru performing pediatric cleft lip repairs while using VIPAAR to connect with expert surgeons in California, United States. Davis et al [53] reported a pediatric neurosurgery case (endoscopic third ventriculostomy) in Ho Chi Minh City, Vietnam, with consultation from a specialist in Birmingham, Alabama, United States. Liu et al [39] described a case of cross-continental telesurgery while also introducing a 3D point-tracking module compatible with the Microsoft HoloLens to accurately track a scalpel’s location. During a skin grafting and fasciotomy of a rabbit model, a surgical trainee wearing the headset in Anhui, China, was able to visualize surgical trajectories drawn by a surgeon in Columbus, Ohio, United States [39]. Van der Putten et al [46] featured the unplanned use of the Microsoft HoloLens 2 to allow a product manager to remotely guide a surgeon through the installation of an implant that would address a complication encountered during total knee arthroplasty. This study uniquely involved a nonsurgeon as the mentoring individual and described the minimal learning curve required for the remote consultant to instruct through the HMD [46].

Andersen et al [59,63], Rojas-Muñoz et al [61], and Zhang et al [68] developed trials to compare the procedural efficiencies of different AR tool setups during telesurgery. Andersen et al [63] compared conventional telestration, in which the hybrid video with the expert’s annotations is displayed on a separate monitor outside the surgical field, with the STAR platform, in which the hybrid video is shown on a tablet directly above the field and the surgeon’s hands. Premedical and medical students were guided through a placement task for would-be laparoscopic ports, followed by abdominal incisions on a model. The STAR-assisted group saw lower placement errors (*P*<.01) and focus shifts away from the operating field (*P*<.001), with time to task completion being slower but not statistically significant (*P*=.17) [63]. A subsequent study by Andersen et al [59] focused on the addition of offline references during a cricothyroidotomy with network limitations. A control group using conventional telestration was compared with a STAR group with access to offline video references showing future steps. Less idle time (*P*<.001) and higher performance scores were observed (*P*<.05 for both raters) in the STAR group [59]. Rojas-Muñoz et al [61] later compared STAR with HMD-STAR, in which the remote viewer’s modifications were displayed on a headset instead of a tablet. The RCT used 2 groups of medical students performing a similar marking and incision task. Across the 2 tasks, the HMD-STAR group had fewer placement errors (*P*<.001 and *P*=.01) and focus shifts (*P*<.001 and *P*<.004) but took more time (*P*<.001 and *P*<.02) [61]. Zhang et al [68] implemented a system (coaxial projective imaging) that allowed a remote mentor’s annotation to directly project onto the local operating field. The study compared the performance of trainees using this system with that of a control group using conventional telestration during a skin cancer surgery simulation. The experimental group demonstrated higher accuracy, shorter operating times, and fewer focus shifts away from the operating field (*P*<.05 each) [68].

### AR in Remote Education

The articles discussed in this section (Multimedia Appendix 3 [32,36,40-42,47-51,58,62,69]) are divided based on nonsurgical contexts, which include clinical skills, autopsy, and sonography, and surgical contexts, which include procedure observation, tool-specific training, simulations, and intraoperative learning. Of note, one-third (6/19, 32%) of the articles that involved education have been described in the previous section and will be briefly mentioned in this section.

#### Nonsurgical

In total, 16% (3/19) of the articles described proof-of-concept studies in which a clinician wore a Microsoft HoloLens 2 to live stream footage of patient examinations during general medicine rounds to remote students. The Microsoft HoloLens 2 allowed students to not only see through the clinician’s eyes using their personal devices but also simultaneously review overlaid 2D patient data and imaging. Rafi et al [47] featured a cardiovascular examination that was remotely spectated by final-year medical students. Using the Microsoft Teams application compatible with the HoloLens, the study measured student engagement in the form of written “chat” comments from students during the session [47]. Bala et al [48] conducted a similar study with remote fourth-year medical students observing a 1-hour session with a patient interview followed by a data interpretation and management planning session. Survey responses from the students found unanimous positive ratings regarding the tool’s impact on accessibility of education, whereas free-response feedback from students, staff, and patients revealed that the technology was a feasible and acceptable method for providing clinical education [48]. Mill et al [49] accommodated 53 fourth-year students split across 3 sessions that each featured a case discussion, bedside review, and debriefing. Survey responses from students and instructors found favorable feedback regarding the teaching quality of the sessions despite one-third of respondents reporting issues with audio and video quality [49].

A few studies (3/19, 16%) integrated AR tools with nonsurgical telementoring. Hanna et al [40] described the use of the Microsoft HoloLens by pathology staff to communicate with an attending pathologist during an autopsy. The headset allowed trainees to view holograms of tissue specimens and web-based procedure manuals, whereas the attending could provide guidance remotely during the procedure [40]. Wang et al [41] introduced the HoloLens to sonography training in a trial comparing an AR-assisted group with an audio-assisted group. Undergraduate and paramedic students were equipped with an AR headset to view the remote mentor’s hand gestures while being instructed on how to perform the right upper quadrant portion of the Focused Assessment with Sonography in Trauma examination. A control group of similar low-experience students wore headphones instead of the headset, with the mentor being able to view their progress through on-site cameras. The results revealed that performance scores were not significantly different between the 2 groups (*P*=.53), although the task completion time was longer for the AR group (*P*=.008) [41]. Mather et al [32] implemented 2 AR-capable HMDs (Vuzix Wrap 1200DX) to create an educational system called “Helping Hands.” A remote instructor’s HMD could capture their hand movements to be overlaid onto the display of a student’s HMD while the student’s hands could be visualized by the instructor. The pilot study involved guiding students through handwashing, with free-text survey responses from students showing favorable impressions of the system [32].

Hess et al [42] described a remote advanced cardiovascular life support simulation using Magic Leap One headsets distributed to second-year medical and physician assistant students. The HMDs used AR to display the holographic simulation apparatus (eg, patients, beds, and monitors) modulated by instructors, thereby allowing students to attend the simulation from their homes. Postsession interviews yielded positive feedback regarding experiential satisfaction and value in practicing communication skills [42].

#### Surgical

AR-capable HMDs also allowed surgical trainees to remotely observe a procedure from the operating surgeon’s perspective. Cofano et al [38], discussed in the previous section, described 2 spine surgeries in which surgical interns and medical students received live commentary from the operating surgeon while also viewing reference models and images.

Vera et al [50] and Patel et al [58] implemented AR for training on the use of surgical tools. Vera et al [50] conducted an RCT using portable laparoscopic training boxes with AR telementoring features to train medical students. An experimental group of students used the AR telementoring platform, in which the instructor’s laparoscopic instruments were superimposed in real time on the student’s monitor during a training session. Compared with a control group that was mentored in person, the AR telementoring group had significantly faster skill acquisition (*P*<.001) and more completed attempts during a posttraining suturing task (*P*=.02) [50]. Patel et al [58] used Proximie to remotely teach medical students how to use robotic surgery tools (da Vinci Skills Simulator), with postexperience Likert surveys showing positive ratings for ease of use and quality of the audio-video feed.

Other articles (4/19, 21%) discussed surgical education performed through simulated procedures. Andersen et al [63] and Rojas-Muñoz et al [61] both featured trials using medical students for abdominal incision tasks and have been discussed in the previous section. Another study by Rojas-Muñoz et al [62] compared HMD-STAR with textbook review for leg fasciotomies performed by medical students and surgical residents. The HMD-STAR group could receive verbal guidance and annotations from a remote mentor, whereas the control group performed the procedure with independent review of the procedure beforehand. When comparing both groups, the HMD-STAR group had a 10% higher weighted individual performance score (*P*=.03) with 67% fewer errors (*P*=.04) and no significant difference between task completion times [62]. Stone et al [69] introduced a novel training system that allowed for the remote instruction of a transperineal prostate biopsy and rectal spacer placement on an anatomical model. The system involved a pair of HMDs that allowed users to view ultrasound imaging and the procedural field simultaneously. The students observed the mentor perform the procedure before practicing on the model with remote guidance. Both learners and instructors reported that the displayed images were adequate for the procedures and that the HMDs did not affect performance negatively [69].

Education in the form of intraoperative telementoring was featured in 21% (4/19) of the studies. A study by Armstrong et al [36] entailed a case report in which a junior resident wore Google Glass while performing a delayed primary closure of a plantar defect under the supervision of a remote attending surgeon. Accessing the audio-video feed through Google Hangouts, the attending could provide verbal feedback and use a mouse on their computer to point to items seen by the Glass [36]. Ponce et al [51] implemented a VIP platform for a pilot study in which surgical residents performed arthroscopic shoulder procedures under the remote mentorship of an attending surgeon. The platform was similar to the VIPAAR platform described previously: video of the operating site was viewed by a remote mentor, allowing the mentor to create annotations or superimpose their own hand and instruments over the video feed. A monitor at the operating site showed the hybrid video to the residents to allow for real-time feedback. Survey responses from those involved showed positive ratings for ease and utility of the tool in anatomical learning, with all in agreement that the system did not compromise safety [51]. Vyas et al [52] and Davis et al [53] were described previously in the context of global surgery, but both demonstrated examples of long-distance surgical training. Mentoring surgeons in the overseas curriculum described by Vyas et al [52] evaluated the local mentees on various aspects of cleft lip repair following both in-person and telementored surgeries. In-person procedures preferentially improved intraoperative decision-making (*P*<.001) and repair principles (*P*<.001), whereas remote sessions preferentially improved understanding of anatomy (*P*<.01) and increased procedural efficiency (*P*<.001) [52].

#### Evaluation of AR-Assisted Remote Tasks

Of the 39 studies, 21 (54%) had a comparative design, with 6 (15%) being RCTs. Most studies (23/39, 59%) gathered data through user feedback surveys or interviews, 70% (16/23) of which used numerical Likert scales. Tables 2 and 3 summarize the variables measured and discussed across the articles in nonsurgical and surgical tasks, respectively.

**Table 2:** Range of variables studied in augmented reality–assisted nonsurgical tasks.

Variable and subcategory			Study, year
**Objective**			
	Performance score	Wang et al [41], 2017 Rigamonti et al [44], 2021	
	Time for task	Chinthammit et al [31], 2014 Wang et al [41], 2017	
	Accuracy	Chinthammit et al [31], 2014 Follmann et al [33], 2019	
	Reliability across task performers	Broach et al [34], 2018 Kaylor et al [37], 2019 Borresen et al [67], 2022	
	Patient complications	Hill [43], 2022	
**Subjective efficacy**			
	Usefulness	Chinthammit et al [31], 2014 Ponce et al [54], 2016 Wang et al [41], 2017 Broach et al [34], 2018 Carbone et al [65], 2018 Mather et al [32], 2018 Follmann et al [33], 2019 Ramsingh et al [64], 2022 Mill et al [49], 2021 Rigamonti et al [44], 2021 Hess et al [42], 2022	
	Improved patient care	Martin et al [45], 2020	
	Improved performance	Chinthammit et al [31], 2014 Wang et al [41], 2017	
	Efficacy in communication	Wang et al [41], 2017 Borresen et al [66], 2019 Ramsingh et al [64], 2019 Martin et al [45], 2020 Bala et al [48], 2021 Mill et al [49], 2021 Hess et al [42], 2022	
	Superiority to other communication methods	Ponce et al [54], 2016 Wang et al [41], 2017	
**Feasibility**			
	Ease of use	Wang et al [41], 2017 Broach et al [34], 2018 Carbone et al [65], 2018 Mather et al [32], 2018 Borresen et al [66], 2019 Martin et al [45], 2020 Mill et al [49], 2021 Rigamonti et al [44], 2021 Hess et al [42], 2022	
	Interference in task	Broach et al [34], 2018 Carbone et al [65], 2018 Mill et al [49], 2021	
	Comfort of device	Mather et al [32], 2018 Martin et al [45], 2020 Rigamonti et al [44], 2021	
**Acceptability**			
	Satisfaction	Ponce et al [54], 2016 Borresen et al [66], 2019 Bala et al [48], 2021 Mill et al [49], 2021 Hess et al [42], 2022	
	Favorability and interest	Mather et al [32], 2018 Rigamonti et al [44], 2021 Mill et al [49], 2021 Rafi et al [47], 2021 Hess et al [42], 2022	
	Trust in use	Carbone et al [65], 2018	
	Graphics quality	Carbone et al [65], 2018 Borresen et al [66], 2019 Ramsingh et al [64], 2019 Bala et al [48], 2021 Mill et al [49], 2021	
**Other measures**			
	Efficacy of device training and instructions	Chinthammit et al [31], 2014 Broach et al [34], 2018 Borresen et al [66], 2019 Hess et al [42], 2022	
	User safety and PPE^a^ use	Martin et al [45], 2020	

^a^PPE: personal protective equipment.

The most common objective variable measured was time to task completion, with other common variables being task performance and procedure-related complications. Several studies (7/39, 18%) on the topics of telerehabilitation and telesurgery found no difference in task time compared with non–AR-assisted conditions, whereas Vera et al [50] found that less time was needed when using AR for laparoscopic training. In contrast, Follmann et al [33], Wang et al [41], and Ponce et al [35] found that AR increased the time needed compared with non-AR conditions for triage assessment, ultrasound examination, and shoulder replacement, respectively.

Performance, measured using scoring systems or frequency of errors, was seen to improve with AR use in telesurgery tasks described by Vera et al [50], Rojas-Muñoz et al [60,62], and Andersen et al [63], whereas Wang et al [41] and Chinthammit et al [31] found no difference when comparing with non-AR groups in tele-ultrasonography and a telerehabilitation-related task, respectively. When comparing AR-assisted tools with each other, as done by Andersen et al [63] and Rojas-Muñoz et al [61], improved performance was observed as AR-enhanced displays were brought closer to the eyes of the user. AR’s impact on resource use was different based on context, with Martin et al [45] finding that AR-assisted rounding during COVID-19 saved PPE, whereas El-Asmar et al [56] and Hassan et al [55] found no significant increases in general anesthesia use in aquablation procedures or contrast use for neuroradiological interventions, respectively.

Subjective measures included in the studies were related to device efficacy, feasibility, and acceptability. All studies that examined feedback-related efficacy, such as the perceived “usefulness” of AR assistance, reported a majority of positive ratings. However, when comparing the ratings of AR-assisted conditions with those of non-AR conditions, Wang et al [41] and Chinthammit et al [31] notably found no differences. Studies that measured ratings for feasibility, such as ease or comfort of use, also found a majority of positive responses. The lowest percentage of positive Likert-scale ratings for ease was observed in first responders in the study by Broach et al [34] in the context of using Google Glass for triage assessments, with 64% (9/14) of users giving a rating of 4 out of 5 or higher. Studies that examined acceptability ratings, such as experience satisfaction or interest, similarly found a consistent majority of positive ratings from AR users. Further study into potential differences between patient and provider ratings may be warranted as Ponce et al [54] found that postoperative patients using a virtual presence–type mobile app were more likely to be satisfied with the overall experience (average rating of 4.6 vs 4.2 out of 5; *P*<.05) and view the virtual interaction as superior to email and SMS text messaging (4.7 vs 4.4 out of 5; *P*<.05) than surgeons.

**Table 3:** Range of variables studied in augmented reality–assisted surgical tasks.

Variable and subcategory			Study, year
**Objective**			
	Performance	Vera et al [50], 2014 Andersen et al [63], 2017 Vyas et al [52], 2020 Andersen et al [59], 2019 Rojas-Muñoz et al [61], 2019 Rojas-Muñoz et al [60], 2020 Rojas-Muñoz et al [62], 2020 Zhang et al [68], 2022	
	Procedural efficiency	Andersen et al [63], 2017 Andersen et al [59], 2019 Rojas-Muñoz et al [61], 2019 Zhang et al [68], 2022	
	Procedure time	Ponce et al [35], 2014 Ponce et al [51], 2014 Vera et al [50], 2014 Andersen et al [63], 2017 Rojas-Muñoz et al [61], 2019 Rojas-Muñoz et al [60], 2020 Rojas-Muñoz et al [62], 2020 Hassan et al [55], 2021 El-Asmar et al [56], 2021 Zhang et al [68], 2022	
	OR^a^ resource use	Hassan et al [55], 2021 El-Asmar et al [56], 2021	
	Complications	Ponce et al [35], 2014 Vyas et al [52], 2020 Hassan et al [55], 2021 El-Asmar et al [56], 2021 Van der Putten et al [46], 2022	
	Patient length of stay and rehospitalization rates	El-Asmar et al [56], 2021	
	Skill acquisition	Vera et al [50], 2014	
	Accuracy of device	Andersen et al [63], 2017 Liu et al [39], 2021	
**Subjective efficacy**			
	Usefulness	Ponce et al [51], 2014 Vera et al [50], 2014 Davis et al [53], 2017 Patel et al [58], 2021	
	Efficiency	Rojas-Muñoz et al [61], 2019 Rojas-Muñoz et al [60], 2020	
	User improvement	Vera et al [50], 2014 Vyas et al [52], 2020	
	Procedural safety	Ponce et al [51], 2014 Vyas et al [52], 2020	
**Feasibility**			
	Ease of use	Ponce et al [51], 2014 Rojas-Muñoz et al [61], 2019 Cofano et al [38], 2021 Patel et al [58], 2021	
	Frustration in use	Rojas-Muñoz et al [61], 2019 Rojas-Muñoz et al [60], 2020	
	Interference in task	Ponce et al [35], 2014 Andersen et al [63], 2017	
	Comfort of device	Rojas-Muñoz et al [61], 2019 Cofano et al [38], 2021 Stone et al [69], 2022	
**Acceptability**			
	Satisfaction	Ponce et al [35], 2014	
	Favorability and interest	Vera et al [50], 2014 Rojas-Muñoz et al [61], 2019 Patel et al [58], 2021 Cofano et al [38], 2021	
	Graphics quality	Ponce et al [35], 2014 Ponce et al [51], 2014 Patel et al [58], 2021 Stone et al [69], 2022	
**Other measures**			
	Device latency	Ponce et al [35], 2014 Ponce et al [51], 2014 Davis et al [53], 2017 Stone et al [69], 2022	
	Financial cost	Davis et al [53], 2017	

^a^OR: operating room.

Although most survey- or interview-based studies measured user feedback about AR’s usefulness (15/23, 65%), far fewer studies (2/23, 9%) surveyed AR’s ability to substitute in-person methods in telemedicine. Borresen et al [66], who used the ARTESH system for motor and strength examinations, found an average Likert rating (4/7) from physicians when they were asked whether in-person examinations would have similar results. Surveyed patients similarly had a lower percentage of positive responses (9/15, 60%) compared with other feasibility and acceptability questions when asked about the device’s potential to substitute an in-person examination [66]. In telementoring, the literature shows that AR may have more potential. Patel et al [58], who used Proximie to remotely mentor students in using robotic surgery tools, observed average ratings above 4/5 for utility as an alternative to in-person mentoring.

Across all 39 studies, there were gaps in the longitudinal measurements related to patient outcomes, such as costs, hospitalization course, and quality of life. Only the study by Davis et al [53] analyzed the finances of implementing an AR system, estimating a cost of US $14,900 per calendar year for a VIPAAR system used for telementoring pediatric neurosurgery in Vietnam. Meanwhile, only El-Asmar et al [56] measured postoperative length of stay and 3-month adverse events to find no significant difference between AR and non-AR conditions. Furthermore, there were few studies (2/39, 5%) that focused on the long-term benefits of AR-enabled remote education, such as retention of learned material and performance over an extended period. Chinthammit et al [31] measured the performance of trainees multiple times over a period of a week after training; however, studies that examined nonsurgical skills over longer periods are absent in this review. Vyas et al [52] described a 13-month overseas course in pediatric cleft lip repair; however, the curriculum combined both in-person and remote intraoperative learning sessions rather than comparing the methods.

## Discussion

### Principal Findings

In this scoping review, we discussed 39 studies that used AR in real-time telemedicine and telementoring. From these studies, 20 unique devices and platforms, most of which involved an HMD, were identified and found to have common features such as annotation, graphical references, and the ability to overlay a remote viewer’s hands or tools onto a local user’s screen.

AR builds on the remote examinations of current audio- and videoconferencing tools by enhancing the remote acquisition and exchange of information. AR technology can supply users with visual aids, such as electronic health record data and guidelines, or facilitate communication with specialists trained to identify specific conditions or manipulate diagnostic equipment. Studies on AR-assisted remote patient evaluations took place in both outpatient and inpatient settings, including emergency triage, wound evaluation, and hospital rounding. Although the devices vary across settings, the comparative studies in this review found noninferior accuracy and reliability of AR-assisted remote conditions compared with in-person controls. Devices dedicated to remote musculoskeletal and sonographic examinations were also developed, with positive user ratings. As technology improves and becomes more accessible, the types of examinations that can be performed remotely will expand and allow for greater access to care, especially by patients and providers in low-resource areas.

The research on AR in the remote delivery of medical interventions predominantly focused on surgery, with other treatment modalities such as physical therapy being less studied [70]. Telesurgery implemented AR features such as virtual presence and annotation to facilitate procedural guidance, mentorship, and consultation. The distance of such connections was tested, with several case reports describing AR in the telementoring of operations across continents. Various types of surgical procedures were telementored using AR, including minimally invasive surgery, orthopedic surgery, and pediatric neurosurgery. The literature surrounding AR and telesurgery focused primarily on the technology’s potential to improve procedural efficiency and communication with experts, with the differences across systems for surgical telementoring studies being investigated in more recent articles. From the data available so far, the performance of AR-assisted procedures appears equivalent to other remotely mentored conditions, with limited research suggesting AR’s potential to substitute in-person mentorship without sacrificing resources or short-term outcomes. Although such findings require further validation, AR plays a promising role in enabling less experienced providers to perform more specialized treatments and avoid the unnecessary transfer of patients across hospital systems. Research on AR-assisted telerehabilitation and psychotherapy could offer insight into whether these nonsurgical treatments could also be effectively performed as remote visits.

Medical education emerged as a common theme underlying AR’s integration into telemedicine and telementoring. In addition to surgery, remote training using AR was studied for clinical skills that are important to inpatient wards and procedures related to pathology and EM. Both surgical and nonsurgical studies implemented AR tools in similar ways, including observational learning, real-time audiovisual feedback, and teaching by demonstration. When in-person mentorship was not possible or inconvenient, the studies showed how AR-enhanced communication allowed distant learners to not just observe but also engage with clinicians and surgeons. Distance learning of surgical and sonographic equipment could also be achieved with AR systems, with few controlled trials thus far indicating noninferior performance results compared with in-person mentoring and superior results compared with unassisted conditions, particularly for less experienced trainees. As the equipment for telecollaboration in medicine becomes readily available, AR technology is expected to advance the sharing of knowledge related to clinical skills, health care devices, and procedural techniques, which directly and indirectly improves patient care outcomes.

Given the early stage of platform development, many studies included in this review (25/39, 64%) used small cohorts of ≤15 patients or participants per study group; although approximately half (21/39, 54%) of the studies included comparative data, far fewer were RCTs. This finding is expected as AR is an emerging technology in medicine, and its use in telemedicine and telementoring is still developing. Existing comparative trials that measured time consumption and performance have so far found that AR-assisted conditions mostly have noninferior results compared with other remote and in-person methods. However, as these studies are heterogeneous in methodology and equipment, dedicated RCTs with standardized designs are needed to understand whether certain AR systems for specific tasks can effectively substitute current remote alternatives or in-person methods. Feedback surveys and interviews were the most common form of data collection observed in this review; subjective variables such as perceived efficacy and ease ratings were commonly measured with consistently positive findings despite the novelty of the technology, suggesting interest across user groups, including providers, trainees, and patients.

### Future Directions

Overall, interventions and learning that require active patient or family caregiver participation (eg, physical therapy, psychotherapy, preventative medicine, chemotherapy, and dialysis) have yet to achieve a similar level of investigation in the space of AR and telemedicine or telementoring as surgery. From the nonsurgical studies included in this review, there is an interest in AR for tele-sonography, EM, and hospital medicine, with many nonsurgical fields yet to be represented. In the realm of patient evaluation, AR’s role is still being separately investigated for electrograms [71], diagnostic procedures related to endoscopy [72], biopsies [73], and urology [74], as well as specialty-specific examinations pertinent to dentistry [75], ophthalmology [76], and dermatology [9], to name a few.

Considering the diversity of medical fields and levels of experience across users, AR-enabled remote interactions are likely to appear in certain settings or users sooner than in others. In the interventional and educational space, studies so far have primarily implemented AR tools for remote consultation between current or future medical professionals. Few trials using untrained individuals for AR-assisted procedural, nonsurgical tasks exist; it would be reasonable to anticipate future research with AR facilitating remote provider-to-patient or provider-to-caregiver interactions in the therapeutic context [77]. To support innovations focused on remote interactions with home caregivers and patients, perspectives from and qualitative studies on these particular end users, rather than solely clinician users, are necessary [22,78].

Although more dedicated RCTs would be needed to assess the efficacy of AR-assisted communication in the treatment setting, AR’s impact on variables related to costs beyond procedure time and outcome measures beyond complication rates remains relatively unexplored. These include hospital-based measurements such as length of stay, equipment costs, and intra- and postoperative pain medication use but also patient-centered variables such as treatment cost, posttreatment functional status, and quality of life. The literature in this review focused mainly on short-term variables, which reveals a lack of longitudinal research that could provide insight into both the long-term outcomes of patients and system-wide effects on productivity and sustainability of AR use. Longitudinal studies with dedicated comparisons, which are likely to increase as devices improve in wearability, usability, and affordability, are also needed to fully understand whether AR could enhance knowledge and skill retention in the remote learning environment.

### Limitations

This review was limited to studies from the last 10 years in 4 medical research databases. Many AR developments in telemedicine and telementoring that exist in the private sector have not been described in published articles and so cannot be systematically evaluated. Notably, a 2019 systematic review of AR in telementoring has also explored additional databases [28]; however, our review included “remote,” “telemedicine,” and “telehealth” as search terms to locate a wider variety of health care tasks that may not rely on equipment or procedures typically associated with “telementoring.” Furthermore, this scoping review placed more emphasis on the diversity of systems using AR and the nonsurgical specialties incorporating them. The small cohorts and predominant collection of subjective data were expected given the novel nature of this intersection and the technology.

Although the potential benefits of AR in telemedicine are promising, the challenges facing this technology are the early stage of research and prototype development for these application contexts and a lack of standardized devices. Outside the original and surgery-specific platforms, most of the hardware observed in this review is available to consumers but at costs that limit widespread use [79]. Furthermore, the adjunct programs and applications used with the hardware greatly varied across the studies. The diversity of tools and their availability could limit the design and generalizability of future trials, especially if the technology is custom-made and difficult to reproduce. Combined with low awareness and a lack of guidelines on how to evaluate AR technology, innovators face difficulty in developing appropriate tools and introducing them into current or even unexplored health care spaces. Future research focusing on the utility and feasibility of AR compared with current technology in the medical setting is paramount, but studies looking into the costs of implementation, user readiness, and user-friendly design are also necessary for successful adoption.

### Conclusions

This scoping review discussed studies that combined AR with real-time telemedicine and telementoring, including patient evaluation, medical intervention, and education. Commonly explored applications for this novel intersection include consultation and procedural guidance, particularly in telesurgery. AR-assisted telecommunication was studied to complement or even improve the capability of remote visits, treatments, and training, but more RCTs are needed to validate task-specific benefits as well as understand the long-term effects for all users. As technology evolves and use at the consumer and industry levels becomes more widespread, research on AR in health care is expected to see larger cohorts, standardized equipment, and more rigorous methods of evaluation. Developing AR tools in medicine must balance user-friendly design with limited research and uptake; such challenges create an opportunity for institutional involvement and a need for perspectives from all those involved in health care, including but not limited to clinicians, caregivers, and patients.

## Appendix

Multimedia Appendix 1 Overview of studies using augmented reality in remote patient evaluation.

Multimedia Appendix 2 Overview of studies using augmented reality in remote medical intervention. Articles denoted with an asterisk (*) also involved educational contexts but are not listed in Multimedia Appendix 3.

Multimedia Appendix 3 Overview of studies using augmented reality in remote medical education. Multimedia Appendix 2 includes additional articles in educational contexts that are also discussed in the Results section.

## Abbreviations

AR: augmented reality
ARTESH: Augmented Reality–based Telerehabilitation System with Haptics
EM: emergency medicine
HMD: head-mounted device
HMD-STAR: head-mounted device with System for Telementoring with Augmented Reality
PPE: personal protective equipment
PRISMA: Preferred Reporting Items for Systematic Reviews and Meta-Analyses
RCT: randomized controlled trial
STAR: System for Telementoring with Augmented Reality
VIP: virtual interactive presence
VIPAAR: virtual interactive presence and augmented reality
VR: virtual reality
WOC: wound, ostomy, and continence
