# Supplementary material for: Augmented Reality in Real-time Telemedicine and Telementoring: Scoping Review
Source: JMIR Mhealth Uhealth. 2023 Apr 18;11:e45464. doi: 10.2196/45464 (PMC10155085; doi:10.2196/45464)
Supplement: Multimedia Appendix 1 [file mhealth_v11i1e45464_app1.docx]

| First author, year | Device at local or remote site | Task (n) | Comparative or control group(s) (n) | Primary findings |
| --- | --- | --- | --- | --- |
| Broach, 2018 [34] | Google Glass, local site | Triage assessment by remote EM^a^ physicians receiving video feed from local responders (44 cases) | EM physicians at local site directly performed assessment (same 44 cases) | - Interrater agreement between same conditions and different conditions not significant (*P=*.41) - Survey responses with high ratings for ease and usefulness - Most rated device would “minimally impede their duties” |
| Follman, 2019 [33] | Recon Jet Smart Glasses, local site | Triage assessment by local responders with remote assistance from an EM physician (38 cases) | First responders without support (240) and first responders with triage algorithm displayed on Smart Glasses (84) | - Control group accuracy 58%, average time 16.6 seconds - Display-assist group accuracy 92% (*P*=.04), average time 37.0 seconds (*P*=.001) - Remote-assist group accuracy 90%, average time 35.0 seconds (both *P*=.01) |
| Ponce, 2016 [54] | Help Lightning mobile app, both sites | Virtual evaluation of 31 patients one week post-procedure by 29 surgeons (1 visit per patient) | N/A | - Mostly positive patient ratings for utility, for overall experience and for superiority over email and text - Less but still mostly positive surgeon ratings for overall experience (*P*< .05) and for superiority over email and text (*P*< .05) - 2 patients with complications in which app delayed care |
| Kaylor, 2019 [37] | Microsoft HoloLens, local site | Wound assessment by local beside nurse with assistance from remote WOC^b^ nurse (21 cases) | Bedside assessments by remote WOC nurse visiting local site and an independent WOC nurse (same 21 cases) | - Intrarater reliability of 98% agreement - Interrater agreement of treatment plan was 100% |
| Hill, 2022 [43] | Microsoft HoloLens 2, local site | Wound assessment by local beside nurse with assistance from remote wound care clinician (12 patients) | Retrospective analysis of similar cases (15) | - Experimental group saw fewer premature dressing removals (*P*=.01), returns to the operating room (*P*=.002) and readmissions (*P*= .004) |
| Borresen, 2019 [66] | ARTESH^c^, both sites | Strength and range of motion evaluation of local patients by remote clinician (15 cases) | In-person examination of local group by independent physician (same 15 cases) | - All clinician volunteers rated above average for ability to evaluate arm strength and for visualizing limb movement but average when asked if in-person and remote exam would have same results - Patients rated positively on ease, satisfaction with experience and potential to substitute in-person exam |
| Borresen, 2022 [67] | (same as above) | (same as above) | (same as above) | - Highest interrater agreement for passive range of motion seen in shoulder abduction and protraction (κ=0.44, CI -0.1 to 1.0) and similarly strength with the addition of elbow flexion (*k*= 0.63, CI 0 to 1.0) - Percent agreement between conditions across all participants ranged from 30% to 100% |
| Rigamonti, 2021 [44] | Microsoft HoloLens 2, local site | Vascular ultrasound examination by local technician shared with remote viewers (1 patient) | N/A | - Interviewees saw greatest potential for AR in education from remote locations - Interviewees saw potential for AR to enhance remote examinations when combined with live data from circulation |
| Carbone, 2018 [65] | Original tele-ultrasound platform, local site | Vascular ultrasound examination by local clinician with remote consultation (12 cases) | N/A | - Consultation regarding device probe position was given 5 times - Surveyed local group considered discussion with remote clinician helpful in reaching diagnosis - Surveyed remote group would trust platform to convey images to express diagnosis in disagreement with local group |
| Martin, 2020 [45] | Microsoft HoloLens 2, remote site | Rounding in COVID-19 wards by senior clinician with team members watching remotely (52 patients) | Traditional clinical rounds in 3 other COVID-19 wards | - Device group with less exposure time by 51.5% and PPE^d^ use by 83.1% across a week (*P*=.002 and *P*=.02) - Majority of users thought device was easy to use, team was safer and had more efficient rounds |

^a^ = emergency medicine

^b^ = wound and ostomy care

^c^ = Augmented Reality based Telerehabilitation System with Haptics

^d^ = personal protective equipment
