# Supplementary material for: Augmented Reality in Real-time Telemedicine and Telementoring: Scoping Review
Source: JMIR Mhealth Uhealth. 2023 Apr 18;11:e45464. doi: 10.2196/45464 (PMC10155085; doi:10.2196/45464)
Supplement: Multimedia Appendix 2 [file mhealth_v11i1e45464_app2.docx]

| First author, year | Device at local or remote site | Task (n) | Comparative or control group(s) (n) | Primary findings |
| --- | --- | --- | --- | --- |
| Chinthammit, 2014 [31] | “Ghostman” (Vuzix Wrap 920AR) at both sites | Remote tutor coached trainees on how to use chopsticks (6 trainees) | Face-to-face training given to control group trainees (6) | - No significant difference for total skill errors (*P*=.54) or time to task completion between training methods (*P*=.75) - No significant difference in Likert scale ratings between training methods |
| Ramsingh, 2019 [64] | iPhones with Vuforia Chalk app at both sites | Ultrasound-guided popliteal nerve block performed in Port-au-Prince, Haiti  with guidance from remote expert in Loma Linda, California, United States (1 case) | N/A | - 5/5 ratings given for communication and ultrasound interpretation - 4/5 from local user for clarity of notations in identifying anatomy, 5/5 for probe placement |
| Rojas-Muñoz, 2020 [60] | STAR^a^ with HMD^b^ at local site | Cricothyroidotomy performed by first responder with remote assistance and evaluation by expert surgeon (20 cases) | Audio-only telemonitoring group (20 cases) | - HMD-STAR^c^ use increased scores across overall population (*P*=.01), low first responder experience (*P*=.01), and low procedure experience groups (*P*=.02) - Completion time not significantly different (*P*=.94) - Surveys show HMD-STAR group had more information to complete task and needed less time |
| *Cofano, 2021 [38] | Moverio BT-300, BT-350, Vuzix Blade, or Microsoft HoloLens at local site | Lumbar arthrodesis performed by surgeons viewed by remote learners (12 cases) | N/A | - Positive feedback regarding ergonomy and comfort of device - Surgeons saw utility of 3D reconstructions and telementoring |
| Hassan, 2021 [55] | Proximie setup at local site | Endovascular neurosurgical procedures performed by local surgical fellow with remote guidance (8 cases) | Cases done with on-site guidance (10) | - No complications in experimental group - No significant difference in contrast use or fluoroscopy times (*P*=.38 and *P*=.85) |
| El-Asmar, 2021 [56] | Proximie setup at local site | Aquablation procedures performed at local site by surgeon with remote guidance (21 cases) | Cases done with on-site guidance (38) | - Experimental group had significantly increased general anesthesia use (*P*<.001) - No significant differences in length of stay, re-hospitalization rates and 3-month adverse events |
| Greenfield, 2018 [57] | Proximie setup at remote site | Hand reconstruction by surgeon in Gaza, Palestine with remote guidance from Beirut, Lebanon (1 case) | N/A | - Platform is reproducible and can benefit areas with little specialized care |
| Ponce, 2014 [35] | VIPAAR^d^ at both sites and Google Glass at local site | Total shoulder replacement by local surgeon in Birmingham, Alabama with remote consultation from Atlanta, Georgia (1 case) | Local surgeon’s usual operating time without remote interaction | - Patient’s function scores significantly improved on two different scales - Operative time is 45 minutes longer than average - Surgeons discuss need for improved battery life, image and sound quality before device can become practical |
| *Vyas, 2020 [52] | VIPAR^e^ setup at remote site | Cleft lip repairs by surgeons in Peru with guidance from California, United States (17 cases) | On-site cleft lip repair operations done by same individuals with in-person guidance (26) | - Only 1 post-op complication following a remote repair at 30-month follow-up - In-person visits preferentially improved preoperative aspects, technique, and intraoperative decision making - Remote sessions better for anatomy understanding and operative efficiency |
| *Davis, 2017 [53] | VIPAR setup at remote site | Pediatric neurosurgery procedure by surgeon in Vietnam with guidance from Birmingham, Alabama, United States (1 case) | N/A | - Video delay ranged 93 to 391 msec relative to audio - Both local and remote surgeons rated system to improve procedural safety and efficiency - Cost per calendar year estimated to be US $14,900 |
| Liu, 2021 [39] | Microsoft HoloLens at local site | Skin grafting and fasciotomy on rabbit model by trainee in Anhui, China with remote guidance from Columbus, Ohio, United States (1 case) | N/A | - Customized scalpel tracker and module allowed for tracking accuracy better than 2 mm - Overall deviation between tracked and guided position on flat surface is less than 2.5 mm |
| Van der Putten, 2022 [46] | Microsoft HoloLens 2 at both sites | Unplanned implant insertion during total knee arthroplasty with remote guidance from a remote product manager (1 case) | N/A | - Patient recovered without complications |
| *Andersen, 2017 [63] | STAR at local site | Placement of adhesives on would-be incision ports, followed by abdominal incisions on model by students with remote guidance (10 students) | Group with traditional telestration, where monitor is placed outside of surgical field (10) | - Experimental group had lower placement error (*P*<.01) and less focus shifts (*P*<.001) - Experiment group was slower but not significant (*P*=.17) |
| *Andersen, 2019 [59] | STAR at local site | Cricothyroidotomy performed by untrained students with remote assistance by expert and access to offline references of future steps (10 students) | Group with traditional telestration (10) | - Idle time ratio for STAR group was 48% less (*P*<.001) - STAR group had 26% less recall error (*P*=0.04) - Performance of STAR group was rated 10% higher (two raters, both *P*<0.5) |
| *Rojas-Muñoz, 2019 [61] | STAR with HMD at local site | Anatomical marking and abdominal incisions on model by medical students with remote guidance (12 students) | Group with STAR but without HMD (8) | - HMD group had fewer placement errors (*P*<.001; *P*=.01) and focus shifts (*P*<.001, *P*=.004) and took more time (*P*<.001, *P*=.01) - Control group had several tablet collisions (3.8 to 4.8 times across two tasks) - Mostly positive survey ratings for ease, efficiency, and usefulness but with concerns about comfort and limited field of vision |
| Zhang, 2022 [68] | Co-axial projective imaging system at local site | Skin cancer surgery simulation performed by untrained user in Anhui, China with remote assistance from expert in Columbus, Ohio, United States (3 trainees) | Group with traditional telestration (3) | - Experimental group was more accurate, quicker and had less focus shifts (each *P*< .05) |

^b^ = head mounted device

^a^ = System for Telementoring with Augmented Reality

^c^ = head mounted device with System for Telementoring with Augmented Reality

^d, e^ =Virtual Interactive Presence and Augmented Reality
