# Supplementary material for: Augmented Reality in Real-time Telemedicine and Telementoring: Scoping Review
Source: JMIR Mhealth Uhealth. 2023 Apr 18;11:e45464. doi: 10.2196/45464 (PMC10155085; doi:10.2196/45464)
Supplement: Multimedia Appendix 3 [file mhealth_v11i1e45464_app3.docx]

| First author, year | Device at local or remote site | Task (n) | Comparative or control group(s) (n) | Primary findings |
| --- | --- | --- | --- | --- |
| Rafi, 2021 [47] | Microsoft HoloLens 2 at local site | Patient examination during general medicine rounds by geriatrics consultant with remote student spectators (1 patient) | N/A | - 146 chat messages from 19 students during session |
| Bala, 2021 [48] | Microsoft HoloLens 2 at local site | Patient examination during teaching wards by clinician with remote student spectators (2 patients) | N/A | - Positive student ratings for enjoyability, educational use and ability to communicate with clinician - Positive comments regarding feasibility and efficacy of tool for clinical education |
| Mill, 2021 [49] | Microsoft HoloLens 2 at local site | Discussion and bedside teaching rounds by instructor with remote student spectators (3 rounds) | N/A | - Positive ratings from students and instructors on quality of sessions - Approximately a third of students and instructors reported audio or video quality issues - Favorable responses from patients with no negative effects on interpersonal communication |
| Hanna, 2018 [40] | Microsoft HoloLens at local site | Autopsy performed by pathology staff with remote supervision (1 case) | N/A | - Successful autopsy with remote attending finding setup convenient |
| Wang, 2017 [41] | Microsoft HoloLens at local site | Right upper quadrant portion of the FAST^A^ exam done by student mentees with remote guidance (12 students) | Group with headphones instead of HMD^b^ (N/A) | - Scores not significantly different between two groups (*P*=.53) - Completion time significantly longer in experimental group (*P*=.01) - Experimental group gave favorable scores for ease and usefulness - Mentor rated control group as easier to guide |
| Mather, 2018 [32] | Vuzix Wrap at both sites | Handwashing procedure by local student mentee with remote guidance (17 students) | N/A | - Student reported tool as useful and connectivity adequate - Comments that tool would be useful when in-person teaching not available |
| Hess, 2022 [42] | Magic Leap One at remote sites | Advanced cardiovascular life support simulation managed by remote students and a facilitator at local site (18 students) | N/A | - Positive feedback regarding acceptability and teaching communication - Suggestions for increased realism and training with tools prior to session |
| Vera, 2014 [50] | ART^c^ platform setup at local site | Laparoscopic peg transfer task by medical students with remote supervision (9 students) | Group with traditional in-person mentoring (9) | - Significantly faster skill acquisition in ART group (*P*<.001) - No significant difference in critical or general errors per attempt - ART group significantly faster during post-training (*P*=.01) and able to complete more attempts (*P*=.02) - 89% of surveyed thought ART was effective |
| Patel, 2021 [58] | Proximie setup at local site | Use of robotic surgery tools by trainees with remote supervision (21 trainees) | N/A | - Ratings with high averages for ease, quality of video and audio, willingness to use again, utility as alternative to in-person teaching |
| Rojas-Muñoz, 2020 [62] | STAR^d^ with HMD at local site | Leg fasciotomy on model by medical trainee with remote supervision (10 trainees) | Group of medical trainees with only independent review of procedure beforehand (10) | - STAR group with greater scores and fewer errors with significance in lower experience group (*P*<.01 and *P*<.001) - No significant difference in task completion times - Self-reported confidence increased in STAR group but not in control |
| Stone, 2022 [69] | Original remote training platform at both sites | Transperineal prostate biopsy done by student with remote guidance and insertion of a rectal space by a student in Pittsford, New York with remote guidance from Scottsdale, Arizona (1 case each) | N/A | - All participants reported that platforms’ images were adequate, headsets did not negatively impact performance and that communication software was intuitive |
| Armstrong, 2014 [36] | Google Glass at local site | Delayed primary closure of plantar defect by resident with remote guidance (1 case) | N/A | - Tool has potential to increase efficiency and patient safety while reducing costs - Lack of available applications and protections for patient information limit implementation |
| Ponce, 2014 [51] | VIPAAR^e^ setup at local site | Arthroscopic shoulder procedure done by resident with remote guidance (15 cases) | Operative times from non-assisted procedures | - Operative time with tool use did not change significantly (*P*=.09) - Both surgeon and residents rated for ease and utility of tool in highlighting anatomy - All those surveyed rated system to not interfere with safety |

^a^ = Focused Assessment with Sonography in Trauma

^b^ = head-mounted device

^c^ = Augmented Reality Telementoring

^d^ = System for Telementoring with Augmented Reality

^e^ = virtual interactive presence and augmented reality
